# Male-Dominant Effects of Chd8 Haploinsufficiency on Synaptic Phenotypes during Development in Mouse Prefrontal Cortex

**DOI:** 10.1021/acschemneuro.3c00690

**Published:** 2024-04-01

**Authors:** Robert A. Ellingford, Mizuki Tojo, M. Albert Basson, Laura C. Andreae

**Affiliations:** † Centre for Developmental Neurobiology, Institute of Psychiatry, Psychology & Neuroscience, King’s College London, London SE1 1UL, U.K.; ‡ Centre for Craniofacial & Regenerative Biology, King’s College London, London SE1 9RT, U.K.; § MRC Centre for Neurodevelopmental Disorders, King’s College London, London, U.K.

**Keywords:** Chd8, Sex difference, ASD, Synaptic neurotransmission, Development, Neuronal structure, Mouse models

## Abstract

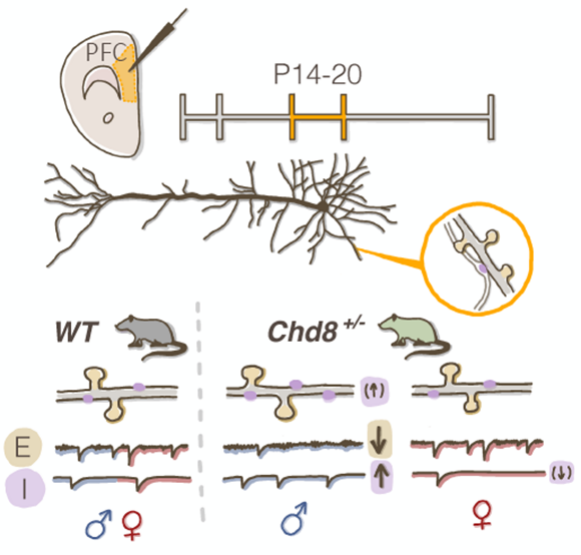

*CHD8* is a high penetrance, high confidence risk gene for autism spectrum disorder (ASD), a neurodevelopmental disorder that is substantially more prevalent among males than among females. Recent studies have demonstrated variable sex differences in the behaviors and synaptic phenotypes of mice carrying different heterozygous ASD-associated mutations in *Chd8*. We examined functional and structural cellular phenotypes linked to synaptic transmission in deep layer pyramidal neurons of the prefrontal cortex in male and female mice carrying a heterozygous, loss-of-function *Chd8* mutation in the C57BL/6J strain across development from postnatal day 2 to adulthood. Notably, excitatory neurotransmission was decreased only in *Chd8*^*+/–*^ males with no differences in *Chd8*^*+/–*^ females, and the majority of alterations in inhibitory transmission were found in males. Similarly, analysis of cellular morphology showed male-specific effects of reduced *Chd8* expression. Both functional and structural phenotypes were most prominent at postnatal days 14–20, a stage approximately corresponding to childhood. Our findings suggest that the effects of *Chd8* mutation are predominantly seen in males and are maximal during childhood.

## Introduction

Autism spectrum disorder (ASD) is a neurodevelopmental disorder affecting social communication with characteristic restrictive or repetitive behaviors. Over the years, diagnosis of ASD has become increasingly common, with recent studies reporting a 2.3% prevalence,^1^ where boys are around four times more likely to be diagnosed than girls.^1−3^ While this male bias in prevalence may partly be attributable to the differences in behavioral manifestations across the sexes,^4,5^ there have also been a number of biological explanations proposed. These broadly relate to either ASD risks linked to male-specific genetic risks and hormones,^6−8^ or to the female protective effect.^9,10^ Studies across multiple disciplines have converged on the notion that sexually dimorphic liability thresholds for ASD are established through the combined effects of risk potentiation in males and attenuation in females.

Perhaps partly due to the increased incidence of ASD in males, pathophysiological mechanisms underlying this disorder have been studied predominantly in males.^11^ However, it is increasingly recognized that studies should draw comparisons across the sexes.^12^ Indeed, there seem to be clear differences in gene expression,^13^ brain structure and connectivity,^14^ and behavior^15,16^ between sexes in several animal models of high confidence ASD risk genes. Recent research has further investigated sexual dimorphism in the context of a specific ASD risk gene, *CHD8*. *CHD8* is one of the highest confidence ASD risk genes and encodes a chromatin remodeling factor, so that its main cellular function is therefore assumed to be in regulating gene expression.^17,18^ Reduction in *CHD8* function has been associated with alterations to cell cycle regulation, neuronal development, immune signaling, and metabolism.^19^ As *CHD8* mutations appear to disproportionately affect males,^18,20^ previous studies have generally focused on characterizing the genetic effects solely in males. Studies have demonstrated that mice with heterozygous mutations or knockdowns in *Chd8* present phenotypic characteristics that resemble autistic individuals with these mutations, including macrocephaly,^21,22^ increased functional connectivity in sensory processing brain regions,^21^ and other core behavioral symptoms of ASD.^21,23^

To explore the sex differential effects of reduced *CHD8* expression, Jung et al. investigated behavioral, synaptic and neuronal phenotypes in a heterozygous *Chd8* mouse line carrying a human ASD-associated mutation (*Chd8*^*+/N2373K*^).^17^ They found that introducing a social or sensory stressor (e.g., maternal separation) induced behavioral impairments predominantly in the males. Analysis of neuronal activation showed that this male-specific behavioral abnormality was paralleled by increased activity in the hippocampus, prefrontal (PFC) and sensory cortex. In contrast, females displayed suppressed baseline activity such that the increase in activation following maternal separation resulted in neuronal activity that remained within the wild-type range. Sex differences were also seen at the synaptic level in hippocampal neurons, where male *Chd8*^*+/N2373K*^ mice showed decreased miniature inhibitory postsynaptic current (mIPSC) frequency and amplitude, while female mutants displayed increased mIPSC frequency. Interestingly, no sex differences were seen in the superficial layers of the medial PFC (mPFC). Recent work by the same group investigated the phenotypes of mice with a different ASD-associated mutation in *Chd8*, *Chd8*^*+/S62X*^.^24,25^ In mice heterozygous for this mutation, changes affecting repetitive and anxiety-like behaviors were found in both sexes in adults, whereas only male juvenile mice exhibited behavioral alterations.^24^ Interestingly, sex differential synaptic changes to excitatory transmission in CA1 neurons of the hippocampus were seen in juveniles.^25^ Together, these studies suggested that sexually dimorphic behaviors and synaptic phenotypes can differ substantially in mice with different *Chd8* mutations. However, a systematic examination of sexual dimorphism in excitatory and inhibitory synapses across multiple developmental stages to identify a potential key window has not been done, nor have the inputs into the key output neurons of the mPFC been examined.

Changes in neuronal activation levels and synaptic transmission often indicate disrupted excitation and inhibition balances in the brain. Excitation–inhibition (E-I) imbalance is hypothesized to represent a characteristic feature of ASD.^26,27^ We have previously found altered synaptic development and homeostatic mechanisms in the mPFC in *Chd8* haploinsufficient mice.^28^ Notably, reduced expression of *Chd8* led to a decrease in excitatory transmission and increase in inhibitory transmission which was maximal between postnatal day 14 and 20, a developmental period roughly corresponding to childhood.

Given the strong implication of the cortical deep-layer pyramidal neurons in ASD etiology,^29,30^ we characterized sexually dimorphic functional neuronal features of layer V/VI mPFC neurons in *Chd8* heterozygous C57BL/6J mice during the developmental period. As we have previously shown that changes in excitatory and inhibitory transmission in *Chd8* heterozygous mice change dynamically throughout development,^28^ we reanalyzed this data in a sex-specific manner and added an additional developmental stage to establish whether reduced *Chd8* expression leads to sex-specific changes in functional characteristics in this mouse model. Finally, we focus on a developmental time window that showed the most significant functional changes in the males and looked at whether sex differences were present in the structural features. Notably, most functional and structural synaptic changes were driven by or stronger in males versus females.

## Results and Discussion

### Male Predominant Effects on Excitatory and Inhibitory Synaptic Transmission in *Chd8*^*+/–*^ C57BL/6J Mice

**Figure 1:**
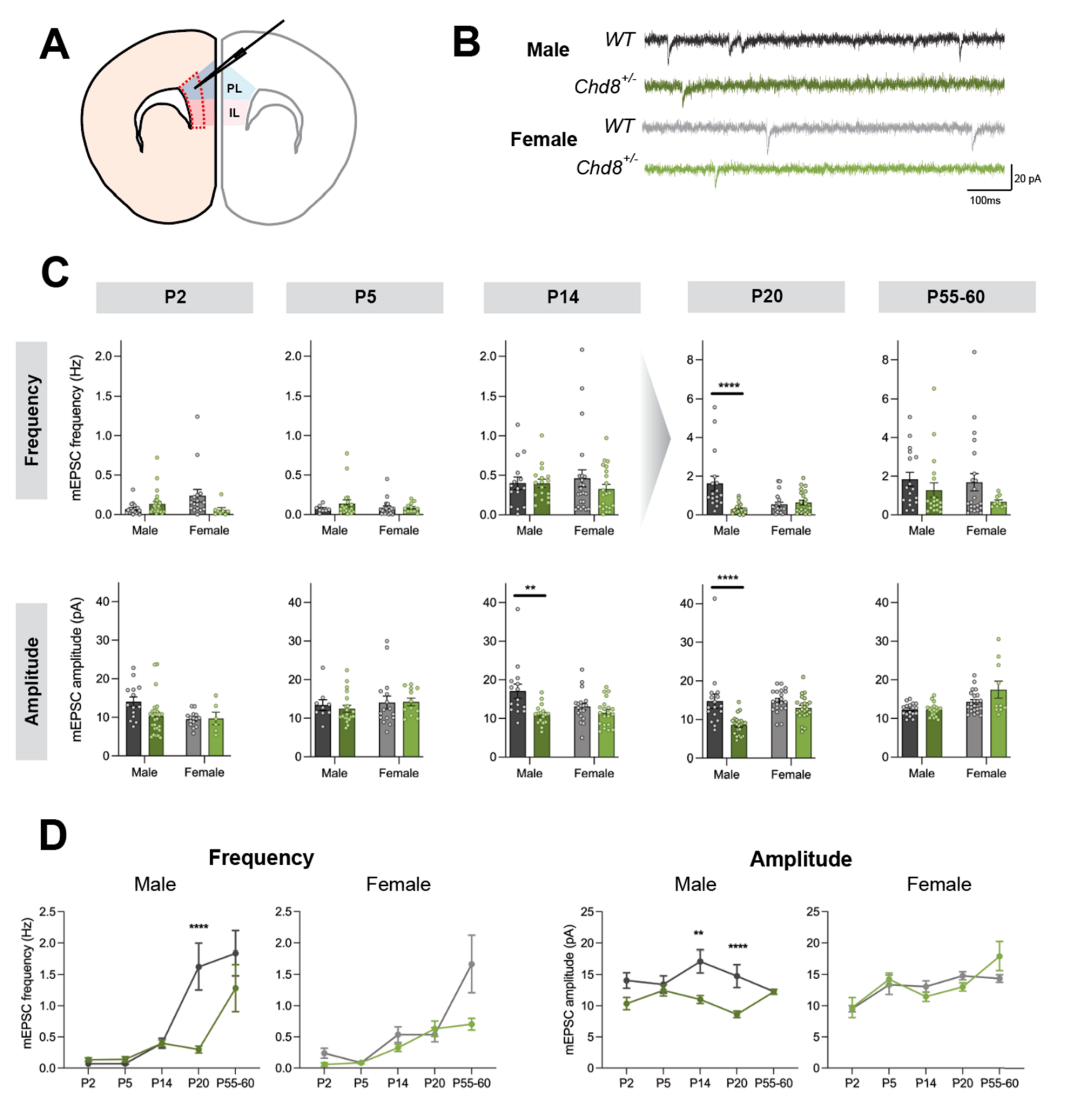
Excitatory transmission in the PFC of *Chd8* heterozygotes is significantly altered only in males. (A) Schematic illustrating coronal *ex vivo* brain slice. Area bound by red dashed border indicates layer V/VI of the mPFC where neurons were recorded; PL – prelimbic cortex, in blue, IL – infralimbic cortex, in pink. (B) Representative mEPSC recordings from deep layer pyramidal neurons in wildtype (WT) and *Chd8*^*+/–*^ male and female mice at postnatal day 20 (P20). (C) *Chd8*^*+/–*^ neurons in males show significantly decreased mEPSC frequency at P20 (*p* < 0.0001) and significantly decreased amplitude at P14 (*p* = 0.001) and P20 (*p* = 0.0002). Gray arrow represents change in scale. (D) Developmental trajectory in males and females. **p < 0.01, ****p < 0.0001. For descriptive and test statistics for all analyses, please refer to Supplementary Tables S1 and S2 in the Supporting Information.

We first investigated whether *Chd8* haploinsufficiency leads to sexually dimorphic effects on synaptic transmission within the deep layers of the PFC, focusing on the prelimbic and infralimbic cortex (Figure 1A).

We examined levels of excitatory neurotransmission across development in the *Chd8*^*+/–*^ mice over a time period between postnatal day 2 (P2) and adult stages (P55–60), including infancy (P5) and childhood (P14, P20). Whole-cell voltage-clamp recordings of miniature excitatory postsynaptic currents (mEPSCs) from deep layer pyramidal neurons in PFC brain slices (Figure 1B) revealed that male *Chd8*^*+/–*^ neurons showed significantly decreased mEPSC amplitude at P14, and decreased mEPSC frequency and amplitude at P20, with no other significant changes at any other developmental stages (Figure 1C, D). In contrast, there were no differences between *Chd8*^*+/–*^ and WT neurons in the females (Figure 1C, D). Furthermore, there were no significant differences in these measures in adult mice, which is suggestive of compensatory mechanisms. We conclude that reduced *Chd8* expression leads to decreased levels of excitatory synaptic transmission only in juvenile males.

**Figure 2:**
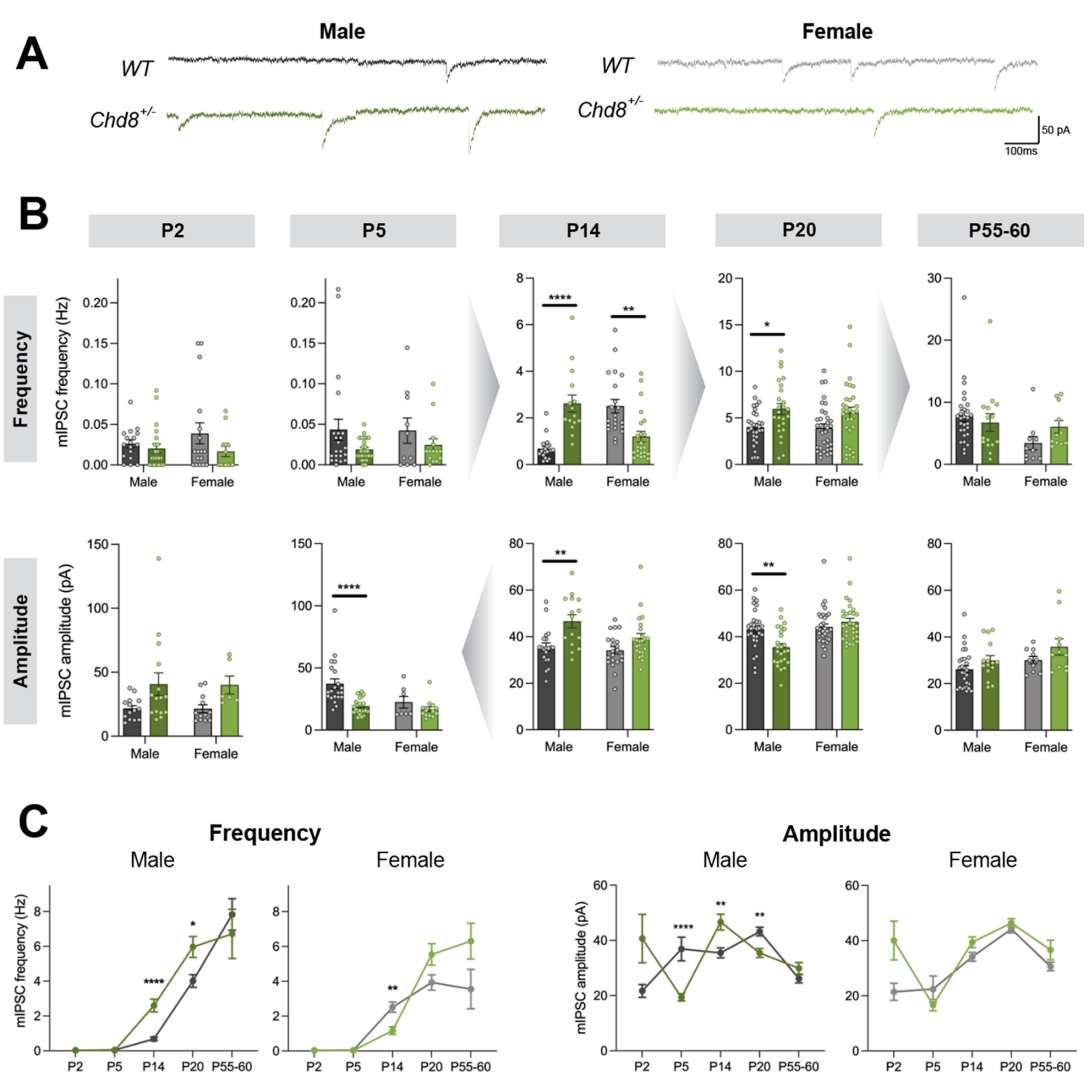
Altered inhibitory transmission in the PFC of Chd8 heterozygotes predominantly in males. (A) Representative mIPSC recordings from deep layer pyramidal neurons in P20 male and female mice. (B) *Chd8*^*+/–*^ neurons in males show significantly increased mIPSC frequency at P14 (*p* < 0.0001) and P20 (*p* = 0.0499), and a significant decrease in females at P14 (*p* = 0.0011). *Chd8*^*+/–*^ neurons in males show significantly decreased mIPSC amplitude at P5 (*p* < 0.0001), significantly increased amplitude at P14 (*p* = 0.0024), and significantly decreased amplitude at P20 (*p* = 0.004). Gray arrows represent changes in scales. (C) Developmental trajectory of inhibitory synaptic transmission in males and females. *p < 0.05, **p < 0.01, ****p < 0.0001.

Recordings of miniature inhibitory postsynaptic currents (mIPSCs) (Figure 2A) across the same spectrum of developmental stages revealed a more complex picture. In the males, no changes were seen at P2, but male *Chd8*^*+/–*^ neurons displayed significantly decreased mIPSC amplitude as early as P5, a time when GABA is likely to be depolarizing.^31^ By P14, when GABA is inhibitory, males have increased mIPSC frequency and amplitude (Figure 2B, C). The increase in mIPSC frequency persists at P20, but by this stage, mIPSC amplitude is decreased in the *Chd8*^*+/–*^ neurons, and there were no significant differences in the adult (Figure 2B, C). While it is not clear what causes these complex changes, it is possible that the interaction between the developmental switch from depolarizing to hyperpolarizing GABA and altered homeostatic responses in *Chd8*^*+/–*^ PFC neurons^28^ may be partly responsible. In contrast, in the females, mIPSC frequency was transiently reduced at P14 but there were no significant differences at any other time points (Figure 2B, C). Overall, these data suggest that *Chd8* heterozygosity is linked to a pronounced change in inhibitory transmission levels predominantly in young and juvenile males.

Taken together, it is clear that a majority of significant changes resulting from *Chd8* haploinsufficiency in both excitatory and inhibitory neurotransmission were seen in males. As would be expected from the fact that there are relatively few synapses early on in development (i.e., P2–P5),^32,33^ we observed very low levels of neurotransmission before P14, and synaptic changes did not persist into adulthood. While we have previously demonstrated the existence of this critical period in early childhood in which E-I balance is significantly altered in the PFC of *Chd8*^*+/–*^ mice,^28^ the sex-specific analysis of this data reported here indicate that this alteration is largely restricted to males.

### Male-Specific Structural Neuronal Changes Due to Reduced *Chd8* Expression

**Figure 3:**
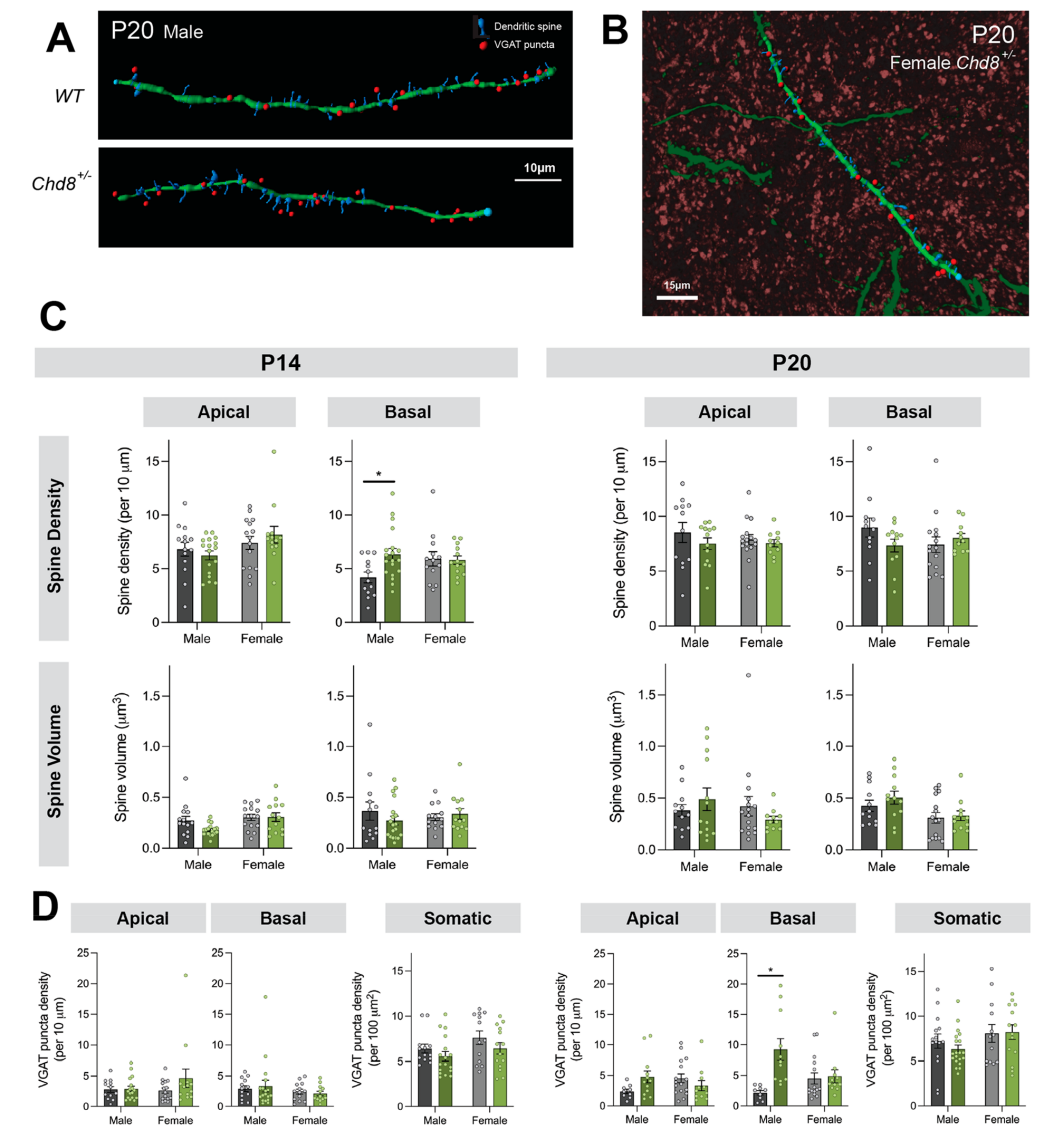
Dendritic spine development and inhibitory synapses of *Chd8+/–* mice. (A) Representative reconstructions of secondary basal dendrites (green) in males at P20, with identified spines (blue) and GABAergic synapses (red dots). (B) Representative reconstruction of female Chd8+/– secondary basal dendrite with dendritic (green) and inhibitory synaptic staining (red puncta) in the background. (C) Analysis of the Spine Densities at P14 and P20. At P14, the basal dendrites show increased spine density in males (*p* = 0.03). At P20, there were no differences in the spine density in both sexes. (D) Analysis of GABAergic Synapses at P14 and P20. At P20, the basal dendrite in males shows a significant increase in inhibitory synapse density (*p* = 0.002). *p < 0.05.

Following on from our observation that the greatest functional changes occur between P14 and P20, we investigated whether the male preponderant alterations in synaptic neurotransmission arose from structural changes to the synapse number. We analyzed the density and volume of dendritic spines, as a proxy for excitatory synapses, as well as the density of inhibitory (i.e., VGAT-positive) synapses, on the secondary apical and basal dendrites of *Chd8*^*+/–*^ neurons (Figure 3A, B). At P14, the only apparent structural effect of *Chd8* haploinsufficiency was increased spine density on basal dendrites in the males (Figure 3C), with no changes in the inhibitory synapse density (Figure 3D). While there were no changes to excitatory spines at P20 (Figure 3C), we did identify a significantly increased number of inhibitory synapses forming onto the basal dendrites, once again only in the males (Figure 3D).

These results extend our previous findings, specifically on the secondary basal dendrites, indicating an increased spine density at P14 and the density of GABAergic synapses at P20, by demonstrating that these changes to these neurons are specific to males. It is likely that *Chd8* haploinsufficiency affected excitatory transmission through changes at the presynapse, specifically a reduction in release probability.^28^ This is reflected in the absence of spine density and volume increases that parallel the increase in mEPSC frequency and amplitude at P20. However, it should be noted that at these developmental stages we were only able to quantify total spine volume rather than spine head volume, which could have contributed to the discrepancy between mEPSC amplitude and spine volume results.

In light of the male-specific increase in mIPSC frequency at P20, a higher inhibitory synapse density on secondary basal dendrites in the *Chd8*^*+/–*^ male mice at this time point suggests that the increased inhibitory transmission may at least in part be related to an increased number of inhibitory synapses onto these dendrites. We have previously established a cell-type-autonomous role for interneurons in driving the observed alterations in inhibitory neurotransmission, whereby inducing *Chd8* haploinsufficiency specifically in interneurons led to a sole increase in mIPSC frequency with no effects on excitatory transmission.^28^ Our results now suggest that *Chd8* haploinsufficiency has a disproportionately greater impact on cortical interneurons in males.

Findings from gene expression analysis across brain regions suggest that it is likely that comparable levels of ASD risk variants are expressed in both sexes, but they are instead modulated through physiological pathways that are sexually dimorphic.^34^ Specifically, while sex differences were not seen in the expression of ASD risk genes themselves within post-mortem cortices, genes that often show elevated expression in post-mortem tissue from autistic brains also showed increased expression in males, particularly sets likely representing downstream effects such as microglia and astrocytic genes.^34^ Conversely, a set of synaptic genes down-regulated in ASD brains was found to be expressed at higher levels in female cortices. Transcriptome analyses in *Chd8*^*+/N2373K*^ mice have further demonstrated greater differences in transcriptomes in female brains, which may suggest a female protective biological mechanism at play.^9^ However, transcriptome analysis of the *Chd8*^*+/S62X*^ mice suggested different age-specific alterations in males versus females.^25^ While a female protective effect is consistent with observations of more disruptive genetic variants found among female autistic individuals,^35^ it is highly likely that sexually dimorphic liability thresholds are established through the combined effects of risk potentiation in males and attenuation in females. Further explorations of the molecular mechanisms underlying these changes at the cellular level may therefore reveal key mechanisms that give rise to sexual dimorphism more generally in *Chd8* haploinsufficiency.

Previous studies on sexual dimorphism in *Chd8* have explored behavioral manifestations of ASD related symptoms, demonstrating abnormal behaviors related to anxiety and emotional regulation and repetitive behaviors. While it is apparent that associations exist between specific *Chd8* mutations and sex differential phenotypic presentations, there appears to be a consistent male-preponderance observed across the different models.^17,24^ A recent study that explored the strain- and sex-specific effects of *Chd8* haploinsufficiency highlighted the large variability in effect sizes of the mutation on key behavioral traits (and brain/body weight) across sexes and strains.^36^ While such findings are largely behavioral, and the extent to which this argument holds for results at the cellular level is unclear, future research should consider the strain and mutation specificity of *Chd8* when exploring their effects on cellular mechanisms.

Our current study has focused on exploring sexual dimorphism in the cellular phenotypes of *Chd8*^*+/–*^ C57BL/6J mice in the mPFC. Our results collectively indicate that reduced *Chd8* expression leads to a male preferential reduction in E-I balance in the mPFC. Additionally, as clinical diagnoses for ASD are made during the first years of development,^37^ the male dominance in the disorder may reflect sexually dimorphic neural changes at this early developmental stage. Indeed, our findings indicate that functional changes in neuronal transmission are present in the early postnatal weeks. Taken together, these findings lend support for the biological explanations of increased liability in males and enhanced protective mechanisms in females, which may account for the over-representation of males in ASD diagnoses made during childhood.

## Methods

Methods are as previously described.^22,28^ Briefly, heterozygous *Chd8* mice (*Chd8*^*+/–*^) expressing approximately 50% less *Chd8* mRNA and protein in the cortex compared to their WT counterparts^22^ were generated by crossing *Chd8*^*+/–*^ with C57BL/6J mice. These *Chd8*^*+/–*^ mice were crossed with Tg(Thy1-EGFP)MJrs/J (*Thy1-GFP-M*) mice for structural analyses. All procedures were performed in accordance with the Animals (Scientific Procedures) Act 1986. Ethical approval was granted by the UK Home Office.

Whole-cell voltage clamp electrophysiological recordings were performed on acute brain slices targeting deep layer (layer V/VI) cortical pyramidal projection neurons. First, action potential firing was inhibited with tetrodotoxin. The mEPSC recordings were conducted by isolating mEPSCs with 10 μM SR-95531 (Gabazine) and using borosilicate glass electrodes filled with K-gluconate internal solution (135 mM K-gluconate, 10 mM KCl, 10 mM HEPES, 1 mM MgCl2, 2 mM Na-adenosine triphosphate (Na_2_ATP) and 0.4 mM Na-guanosine triphosphate (Na_3_GTP)). In contrast, mIPSCs were isolated with 10 μM 2,3-dihydroxy-6-nitro-7-sulfamoyl-benzo[*f*]quinoxaline (NBQX) and 25 μM (2*R*)-amino-5-phosphonovaleric acid (D-APV), and recordings were made using electrodes filled with Cl^–^-loaded internal solution (150 mM CsCl, 1.5 mM MgCl_2_, 0.5 mM EGTA, 10 mM HEPES, 4 mM Na_2_ATP, and 0.4 mM Na_3_GTP). Frequency and amplitude of mEPSCs and mIPSCs were calculated by taking the average of three 60-s trains of spontaneous activity recorded with membrane potential clamped at −70 mV. Finally, traces were analyzed using MiniAnalysis Program 6.0.3 software (Synaptosoft).

For immunohistochemistry, *Chd8*^*+/–*^ mice were crossed with the *Thy1-GFP-M* line, and the offspring perfusion fixed with 4% paraformaldehyde at P14 and P20 and postfixed in PFA for 24 h. These *Chd8*^*+/–*^*;Thy1-GFP-M* brains were sectioned into 100 μm coronal slices by using a Leica VT 1000S vibratome. Selected slices containing the PFC were then permeabilized in PBS supplemented with 1% Triton-X100 (PBS-T) for 4 h and blocked overnight in 3% bovine serum albumin, 10% fetal bovine serum, 0.2 M glycine, in PBS-T, before incubation for 3 h at 4 °C in primary antibodies labeling green fluorescent protein (GFP; 1:1000, chicken anti-GFP, Abcam) and vesicular GABA transporter (VGAT; 1:1000, rabbit anti-VGAT, Synaptic Systems). Slices were subsequently washed and incubated overnight at room temperature in secondary antibodies (goat anti-chicken Fluor488 and goat anti-rabbit Fluor568, 1:2000, Alexa). GFP^+^ layer V/VI pyramidal neurons were imaged using either a Zeiss LSM 800 (P14) or a Nikon A1R point-scanning-confocal microscope (P20) under a 63× or 100× oil immersion objective, respectively. From each animal, the dendrites of at least 4 neurons were imaged, with a single secondary apical and a basal dendrite imaged from each neuron. Quantifications and reconstructions of structural features were performed using IMARIS software (BitPlane), whereby dendrites were fully reconstructed using the Filament Tracer suite, and then total spine volumes algorithmically reconstructed based on the boundaries of the EGFP fluorescent signal. Total spine volume was quantified as at these more developmental stages it was often impossible to clearly demarcate spine head from neck, so this approach resulted in an unbiased assessment of total volume. Imaging and analyses were conducted blind to sample genotypes.

Analyses of the data were performed blind to genotype and treatment using Prism software (GraphPad) (see the Supporting Information). For pairwise comparisons, appropriate statistical tests were conducted depending on the normality of the data set (*t* test for parametric data, Mann–Whitney for nonparametric). Multivariate comparisons were conducted through a two-way ANOVA and a subsequent Tukey’s multiple comparisons test. Data are shown as mean ± standard error of the mean unless otherwise stated.

## Supporting Information Available

Supplementary tables indicating sample sizes, descriptive and test statistics of all statistical analyses (PDF)

## Abbreviations

*CHD8*: Chromodomain Helicase DNA binding factor 8
ASD: Autism Spectrum Disorder
mPFC: Medial Prefrontal Cortex
mEPSC: Miniature Excitatory Postsynaptic Current
mIPSC: Miniature Inhibitory Postsynaptic Current
GABA: Gamma-aminobutyric acid
