# Supplementary material for: Male-Dominant Effects of Chd8 Haploinsufficiency on Synaptic Phenotypes during Development in Mouse Prefrontal Cortex
Source: ACS Chem Neurosci. 2024 Apr 1;15(8):1635–42. doi: 10.1021/acschemneuro.3c00690 (PMC11027092; doi:10.1021/acschemneuro.3c00690)
Supplement: Supplementary file 1 — cn3c00690_si_001.pdf [file cn3c00690_si_001.pdf]

Supporting Information

Male-dominant effects of Chd8 haploinsufficiency on synaptic phenotypes during development in mouse prefrontal cortex

Author List
Robert A. Ellingford[1,2], Mizuki Tojo[1], M. Albert Basson[2,3], Laura C. Andreae*[1,3]

Affiliations
[1] Centre for Developmental Neurobiology, Institute of Psychiatry, Psychology & Neuroscience, King's College London, London, SE1 1UL, UK
[2] Centre for Craniofacial & Regenerative Biology, King's College London, London, SE1 9RT, UK
[3] MRC Centre for Neurodevelopmental Disorders, King's College London, London, UK

Contact
*Corresponding Author: laura.andreae@kcl.ac.uk

Table S1: *Descriptive Statistics*

Comparison (Units)	Sex-Genotype	N (Neurons (animals))	Mean	S.E.M (±)
**P2 mEPSC frequency (Hz)**	M-WT	16 (4)	0.07	0.02
	M-*Chd8*$^{+/-}$	26(6)	0.13	0.03
	F-WT	16 (3)	0.24	0.08
	F-*Chd8*$^{+/-}$	8 (3)	0.06	0.03
**P2 mEPSC amplitude (pA)**	M-WT	14 (4)	14.05	1.3
	M-*Chd8*$^{+/-}$	26(6)	10.35	1.0
	F-WT	15 (3)	9.54	0.5
	F-*Chd8*$^{+/-}$	5 (3)	9.71	1.6
**P5 mEPSC frequency (Hz)**	M-WT	8 (3)	0.07	0.02
	M-*Chd8*$^{+/-}$	20 (3)	0.13	1.0
	F-WT	20 (4)	0.09	0.02
	F-*Chd8*$^{+/-}$	12 (3)	0.09	0.02
**P5 mEPSC amplitude (pA)**	M-WT	9 (3)	13.42	1.4
	M-*Chd8*$^{+/-}$	20 (3)	12.47	0.9
	F-WT	18 (4)	13.37	1.6
	F-*Chd8*$^{+/-}$	12 (3)	14.25	1.0
**P14 mEPSC frequency (Hz)**	M-WT	15 (3)	0.40	0.08
	M-*Chd8*$^{+/-}$	17 (3)	0.40	0.05
	F-WT	19 (3)	0.54	0.02
	F-*Chd8*$^{+/-}$	21 (3)	0.33	0.06
**P14 mEPSC amplitude (pA)**	M-WT	15 (3)	17.09	1.8
	M-*Chd8*$^{+/-}$	17 (3)	11.00	0.6
	F-WT	19 (3)	13.07	0.9
	F-*Chd8*$^{+/-}$	21 (3)	11.46	0.8
**P20 mEPSC frequency (Hz)**	M-WT	17 (3)	1.62	0.4
	M-*Chd8*$^{+/-}$	22 (4)	0.30	0.06
	F-WT	21 (3)	0.54	0.1
	F-*Chd8*$^{+/-}$	22 (4)	0.63	0.1
**P20 mEPSC amplitude (pA)**	M-WT	17 (3)	14.76	1.8
	M-*Chd8*$^{+/-}$	22 (4)	8.62	0.5
	F-WT	21 (3)	14.81	0.7
	F-*Chd8*$^{+/-}$	22 (4)	13.02	0.7
**P55-60 mEPSC frequency (Hz)**	M-WT	17 (3)	1.84	0.4
	M-*Chd8*$^{+/-}$	19 (3)	1.28	0.4
	F-WT	22 (3)	1.67	0.5
	F-*Chd8*$^{+/-}$	10 (3)	0.70	0.09
**P55-60 mEPSC amplitude (pA)**	M-WT	17 (3)	12.28	0.4
	M-*Chd8*$^{+/-}$	19 (3)	12.24	0.4
	F-WT	22 (3)	14.37	0.6
	F-*Chd8*$^{+/-}$	10 (3)	17.92	2.3
**P2 mIPSC frequency (Hz)**	M-WT	17 (4)	0.026	0.005
	M-*Chd8*$^{+/-}$	21 (3)	0.020	0.006
	F-WT	17 (4)	0.039	0.01
	F-*Chd8*$^{+/-}$	13 (3)	0.017	0.006
**P2 mIPSC amplitude (pA)**	M-WT	14 (4)	21.69	2.3
	M-*Chd8*$^{+/-}$	15 (3)	40.65	8.8
	F-WT	12 (4)	21.4	3.1
	F-*Chd8*$^{+/-}$	6 (3)	40.03	7.1
**P5 mIPSC frequency (Hz)**	M-WT	22 (3)	0.043	0.02
	M-*Chd8*$^{+/-}$	26 (4)	0.019	0.003
	F-WT	10 (3)	0.042	0.02
	F-*Chd8*$^{+/-}$	14 (3)	0.024	0.008
**P5 mIPSC amplitude (pA)**	M-WT	19 (3)	36.85	4.3
	M-*Chd8*$^{+/-}$	24 (4)	19.35	1.3
	F-WT	7 (3)	22.44	4.8
	F-*Chd8*$^{+/-}$	13 (3)	16.65	2.1

**P14 mIPSC frequency (Hz)**	M-WT	20 (3)	0.68	0.1
	M-*Chd8*$^{+/-}$	15 (3)	2.60	0.4
	F-WT	21 (3)	2.51	0.3
	F-*Chd8*$^{+/-}$	23 (4)	1.17	0.2
**P14 mIPSC amplitude (pA)**	M-WT	20 (3)	35.48	1.8
	M-*Chd8*$^{+/-}$	15 (3)	46.63	2.9
	F-WT	21 (3)	34.12	1.6
	F-*Chd8*$^{+/-}$	23 (4)	39.41	1.9
**P20 mIPSC frequency (Hz)**	M-WT	29 (4)	4.01	0.4
	M-*Chd8*$^{+/-}$	25 (3)	5.96	0.6
	F-WT	32 (4)	3.93	0.4
	F-*Chd8*$^{+/-}$	30 (4)	5.54	0.6
**P20 mIPSC amplitude (pA)**	M-WT	29 (4)	43.19	1.6
	M-*Chd8*$^{+/-}$	25 (3)	35.40	1.6
	F-WT	32 (4)	44.16	1.3
	F-*Chd8*$^{+/-}$	30 (4)	46.37	1.5
**P55-60 mIPSC frequency (Hz)**	M-WT	28 (5)	7.83	0.9
	M-*Chd8*$^{+/-}$	15 (3)	6.71	1.4
	F-WT	10 (3)	3.55	1.1
	F-*Chd8*$^{+/-}$	11 (3)	6.30	1.0
**P55-60 mIPSC amplitude (pA)**	M-WT	28 (5)	26.11	1.5
	M-*Chd8*$^{+/-}$	19 (3)	29.87	2.1
	F-WT	10 (3)	30.62	1.6
	F-*Chd8*$^{+/-}$	11 (3)	36.60	3.6
**P14 apical spines (per 10 µm)**	M-WT	14 (2)	6.81	0.6
	M-*Chd8*$^{+/-}$	17 (3)	6.24	0.4
	F-WT	15 (3)	7.40	0.6
	F-*Chd8*$^{+/-}$	13 (3)	8.17	0.8
**P14 basal spines (per 10 µm)**	M-WT	13 (2)	4.19	0.5
	M-*Chd8*$^{+/-}$	19 (3)	6.34	0.5
	F-WT	13 (3)	5.92	0.7
	F-*Chd8*$^{+/-}$	13 (3)	5.80	0.4
**P20 apical spines (per 10 µm)**	M-WT	12 (2)	8.53	0.9
	M-*Chd8*$^{+/-}$	13 (3)	7.51	0.5
	F-WT	16 (3)	7.88	0.5
	F-*Chd8*$^{+/-}$	11 (2)	7.56	0.3
**P20 basal spines (per 10 µm)**	M-WT	12 (2)	8.98	0.9
	M-*Chd8*$^{+/-}$	12 (3)	7.35	0.6
	F-WT	15 (3)	7.42	0.7
	F-*Chd8*$^{+/-}$	11 (2)	8.04	0.4
**P14 apical VGAT puncta (per 10 µm)**	M-WT	9 (2)	2.84	0.5
	M-*Chd8*$^{+/-}$	12 (3)	2.93	0.4
	F-WT	15 (3)	2.66	0.4
	F-*Chd8*$^{+/-}$	13 (3)	4.66	1.5
**P14 basal VGAT puncta (per 10 µm)**	M-WT	13 (2)	2.95	0.5
	M-*Chd8*$^{+/-}$	18 (3)	3.36	1.0
	F-WT	18 (3)	2.48	0.4
	F-*Chd8*$^{+/-}$	12 (3)	2.16	0.4
**P14 somatic VGAT puncta (per 10 µm)**	M-WT	13 (2)	6.42	0.5
	M-*Chd8*$^{+/-}$	17 (3)	5.57	0.5
	F-WT	13 (3)	7.62	0.7
	F-*Chd8*$^{+/-}$	14 (3)	6.43	0.6
**P20 apical VGAT puncta (per 10 µm)**	M-WT	9 (2)	2.39	0.4
	M-*Chd8*$^{+/-}$	12 (3)	4.79	1.0
	F-WT	16 (3)	4.53	0.7
	F-*Chd8*$^{+/-}$	11 (2)	3.36	0.8
**P20 basal VGAT puncta (per 10 µm)**	M-WT	9 (2)	2.95	0.5
	M-*Chd8*$^{+/-}$	11 (3)	3.36	1.0
	F-WT	15 (3)	2.48	0.4
	F-*Chd8*$^{+/-}$	11 (2)	2.16	0.4
**P20 somatic VGAT puncta (per 10 µm)**	M-WT	15 (2)	7.21	0.8
	M-*Chd8*$^{+/-}$	20 (3)	6.31	0.4

	F-WT	13 (3)	8.07	1.0
	F-*Chd8*$^{+/-}$	14 (2)	8.20	0.8

Table S2: *Test Statistics*

**Comparison (Units)**		**Tukey's Adjusted P**	**ANOVA**	**F**	**P**
**P2 mEPSC frequency (Hz)**	M-WT vs M-*Chd8*$^{+/-}$	0.71	Interaction	(1, 62) = 5.7	0.02
	M-WT vs F-WT	0.07			
	F-WT vs F-*Chd8*$^{+/-}$	0.14			
	M-*Chd8*$^{+/-}$ vs F-*Chd8*$^{+/-}$	0.75			
**P2 mEPSC amplitude (pA)**	M-WT vs M-*Chd8*$^{+/-}$	0.50	Interaction	(1, 58) = 6.9	0.01
	M-WT vs F-WT	0.43			
	F-WT vs F-*Chd8*$^{+/-}$	0.13			
	M-*Chd8*$^{+/-}$ vs F-*Chd8*$^{+/-}$	0.15			
**P5 mEPSC frequency (Hz)**	M-WT vs M-*Chd8*$^{+/-}$	0.60	Interaction	(1, 56) = 0.86	0.36
	M-WT vs F-WT	> 0.99	Sex	(1, 56) = 0.33	0.57
	F-WT vs F-*Chd8*$^{+/-}$	> 0.99	Genotype	(1, 56) = 0.88	0.35
	M-*Chd8*$^{+/-}$ vs F-*Chd8*$^{+/-}$	0.67			
**P5 mEPSC amplitude (pA)**	M-WT vs M-*Chd8*$^{+/-}$	0.96	Interaction	(1, 53) = 0.18	0.67
	M-WT vs F-WT	0.99	Sex	(1, 53) = 0.81	0.37
	F-WT vs F-*Chd8*$^{+/-}$	> 0.99	Genotype	(1, 53) = 0.08	0.78
	M-*Chd8*$^{+/-}$ vs F-*Chd8*$^{+/-}$	0.75			
**P14 mEPSC frequency (Hz)**	M-WT vs M-*Chd8*$^{+/-}$	> 0.99	Interaction	(1, 72) = 0.63	0.43
	M-WT vs F-WT	0.96	Sex	(1, 72) = 0.005	0.94
	F-WT vs F-*Chd8*$^{+/-}$	0.61	Genotype	(1, 72) = 0.66	0.42
	M-*Chd8*$^{+/-}$ vs F-*Chd8*$^{+/-}$	0.93			
**P14 mEPSC amplitude (pA)**	M-WT vs M-*Chd8*$^{+/-}$	0.001	Interaction	(1, 68) = 4.50	0.04
	M-WT vs F-WT	0.05			
	F-WT vs F-*Chd8*$^{+/-}$	0.67			
	M-*Chd8*$^{+/-}$ vs F-*Chd8*$^{+/-}$	0.99			
**P20 mEPSC frequency (Hz)**	M-WT vs M-*Chd8*$^{+/-}$	< 0.0001	Interaction	(1, 78) = 15.58	0.0002
	M-WT vs F-WT	0.0005			
	F-WT vs F-*Chd8*$^{+/-}$	0.98			
	M-*Chd8*$^{+/-}$ vs F-*Chd8*$^{+/-}$	0.53			
**P20 mEPSC amplitude (pA)**	M-WT vs M-*Chd8*$^{+/-}$	0.0002	Interaction	(1, 78) = 5.15	0.03
	M-WT vs F-WT	< 0.99			
	F-WT vs F-*Chd8*$^{+/-}$	0.69			
	M-*Chd8*$^{+/-}$ vs F-*Chd8*$^{+/-}$	0.007			
**P55-60 mEPSC frequency (Hz)**	M-WT vs M-*Chd8*$^{+/-}$	0.75	Interaction	(1, 64) = 0.29	0.59
	M-WT vs F-WT	0.99	Sex	(1, 64) = 0.82	0.37
	F-WT vs F-*Chd8*$^{+/-}$	0.39	Genotype	(1, 64) = 3.47	0.07
	M-*Chd8*$^{+/-}$ vs F-*Chd8*$^{+/-}$	0.78			
**P55-60 mEPSC amplitude (pA)**	M-WT vs M-*Chd8*$^{+/-}$	> 0.99	Interaction	(1, 64) = 3.71	0.06
	M-WT vs F-WT	0.24	Sex	(1, 64) = 18.36	< 0.0001
	F-WT vs F-*Chd8*$^{+/-}$	0.07	Genotype	(1, 64) = 3.50	0.07
	M-*Chd8*$^{+/-}$ vs F-*Chd8*$^{+/-}$	0.0009			
**P2 mIPSC frequency (Hz)**	M-WT vs M-*Chd8*$^{+/-}$	0.95	Interaction	(1, 64) = 0.97	0.97
	M-WT vs F-WT	0.69	Sex	(1, 64) = 0.31	0.58
	F-WT vs F-*Chd8*$^{+/-}$	0.29	Genotype	(1, 64) = 2.87	0.10
	M-*Chd8*$^{+/-}$ vs F-*Chd8*$^{+/-}$	0.99			
**P2 mIPSC amplitude (pA)**	M-WT vs M-*Chd8*$^{+/-}$	0.10	Interaction	(1, 43) = 7.84	0.01
	M-WT vs F-WT	< 0.99			
	F-WT vs F-*Chd8*$^{+/-}$	0.32			
	M-*Chd8*$^{+/-}$ vs F-*Chd8*$^{+/-}$	< 0.99			
**P5 mIPSC frequency (Hz)**	M-WT vs M-*Chd8*$^{+/-}$	0.17	Interaction	(1, 68) = 4.18	0.045
	M-WT vs F-WT	> 0.99			
	F-WT vs F-*Chd8*$^{+/-}$	0.98			
	M-*Chd8*$^{+/-}$ vs F-*Chd8*$^{+/-}$	0.98			

**P5 mIPSC amplitude (pA)**	M-WT vs M-*Chd8*$^{+/-}$	< 0.0001	Interaction	(1, 61) = 3.14	0.08
	M-WT vs F-WT	0.04	Sex	(1, 61) = 6.56	0.01
	F-WT vs F-*Chd8*$^{+/-}$	0.75	Genotype	(1, 61) = 12.01	0.001
	M-*Chd8*$^{+/-}$ vs F-*Chd8*$^{+/-}$	0.92			
**P14 mIPSC frequency (Hz)**	M-WT vs M-*Chd8*$^{+/-}$	< 0.0001	Interaction	(1, 75) = 41.57	0.0001
	M-WT vs F-WT	< 0.0001			
	F-WT vs F-*Chd8*$^{+/-}$	0.0011			
	M-*Chd8*$^{+/-}$ vs F-*Chd8*$^{+/-}$	0.0013			
**P14 mIPSC amplitude (pA)**	M-WT vs M-*Chd8*$^{+/-}$	0.0024	Interaction	(1, 75) = 2.11	0.15
	M-WT vs F-WT	0.96	Sex	(1, 75) = 4.52	0.04
	F-WT vs F-*Chd8*$^{+/-}$	0.21	Genotype	(1, 75) = 16.59	0.0001
	M-*Chd8*$^{+/-}$ vs F-*Chd8*$^{+/-}$	0.075			
**P20 mIPSC frequency (Hz)**	M-WT vs M-*Chd8*$^{+/-}$	0.0499	Interaction	(1, 113) = 0.11	0.74
	M-WT vs F-WT	< 0.99	Sex	(1, 113) = 0.24	0.62
	F-WT vs F-*Chd8*$^{+/-}$	0.10	Genotype	(1, 113) = 41.57	0.0007
	M-*Chd8*$^{+/-}$ vs F-*Chd8*$^{+/-}$	0.94			
**P20 mIPSC amplitude (pA)**	M-WT vs M-*Chd8*$^{+/-}$	0.004	Interaction	(1, 113) = 10.97	0.001
	M-WT vs F-WT	0.97			
	F-WT vs F-*Chd8*$^{+/-}$	0.70			
	M-*Chd8*$^{+/-}$ vs F-*Chd8*$^{+/-}$	< 0.0001			
**P55-60 mIPSC frequency (Hz)**	M-WT vs M-*Chd8*$^{+/-}$	0.87	Interaction	(1, 60) = 2.27	0.14
	M-WT vs F-WT	0.054	Sex	(1, 60) = 4.19	0.045
	F-WT vs F-*Chd8*$^{+/-}$	0.56	Genotype	(1, 60) = 0.37	0.55
	M-*Chd8*$^{+/-}$ vs F-*Chd8*$^{+/-}$	0.98			
**P55-60 mIPSC amplitude (pA)**	M-WT vs M-*Chd8*$^{+/-}$	0.51	Interaction	(1, 60) = 0.18	0.67
	M-WT vs F-WT	0.58	Sex	(1, 60) = 4.64	0.04
	F-WT vs F-*Chd8*$^{+/-}$	0.42	Genotype	(1, 60) = 4.30	0.04
	M-*Chd8*$^{+/-}$ vs F-*Chd8*$^{+/-}$	0.30			
**P14 apical spines (per 10 µm)**	M-WT vs M-*Chd8*$^{+/-}$	0.90	Interaction	(1, 55) = 1.23	0.27
	M-WT vs F-WT	0.90	Sex	(1, 55) = 4.32	0.04
	F-WT vs F-*Chd8*$^{+/-}$	0.82	Genotype	(1, 55) = 0.03	0.87
	M-*Chd8*$^{+/-}$ vs F-*Chd8*$^{+/-}$	0.12			
**P14 basal spines (per 10 µm)**	M-WT vs M-*Chd8*$^{+/-}$	0.03	Interaction	(1, 54) = 4.30	0.04
	M-WT vs F-WT	0.15			
	F-WT vs F-*Chd8*$^{+/-}$	> 0.99			
	M-*Chd8*$^{+/-}$ vs F-*Chd8*$^{+/-}$	0.88			
**P20 apical spines (per 10 µm)**	M-WT vs M-*Chd8*$^{+/-}$	0.63	Interaction	(1, 48) = 0.35	0.56
	M-WT vs F-WT	0.85	Sex	(1, 48) = 0.26	0.61
	F-WT vs F-*Chd8*$^{+/-}$	0.98	Genotype	(1, 48) = 1.27	0.27
	M-*Chd8*$^{+/-}$ vs F-*Chd8*$^{+/-}$	> 0.99			
**P20 basal spines (per 10 µm)**	M-WT vs M-*Chd8*$^{+/-}$	0.36	Interaction	(1, 46) = 2.68	0.11
	M-WT vs F-WT	0.35	Sex	(1, 46) = 0.40	0.53
	F-WT vs F-*Chd8*$^{+/-}$	0.92	Genotype	(1, 46) = 0.54	0.47
	M-*Chd8*$^{+/-}$ vs F-*Chd8*$^{+/-}$	0.90			
**P14 apical VGAT puncta (per 10 µm)**	M-WT vs M-*Chd8*$^{+/-}$	> 0.99	Interaction	(1, 53) = 1.40	0.24
	M-WT vs F-WT	> 0.99	Sex	(1, 53) = 0.92	0.34
	F-WT vs F-*Chd8*$^{+/-}$	0.31	Genotype	(1, 53) = 1.68	0.20
	M-*Chd8*$^{+/-}$ vs F-*Chd8*$^{+/-}$	0.41			
**P14 basal VGAT puncta (per 10 µm)**	M-WT vs M-*Chd8*$^{+/-}$	> 0.97	Interaction	(1, 52) = 0.26	0.61
	M-WT vs F-WT	> 0.97	Sex	(1, 52) = 1.37	0.25
	F-WT vs F-*Chd8*$^{+/-}$	0.99	Genotype	(1, 52) = 0.003	0.96
	M-*Chd8*$^{+/-}$ vs F-*Chd8*$^{+/-}$	0.62			
**P14 somatic VGAT puncta (per 10 µm)**	M-WT vs M-*Chd8*$^{+/-}$	0.73	Interaction	(1, 59) = 0.42	0.52
	M-WT vs F-WT	0.53	Sex	(1, 59) = 2.99	0.09
	F-WT vs F-*Chd8*$^{+/-}$	0.52	Genotype	(1, 59) = 5.40	0.02
	M-*Chd8*$^{+/-}$ vs F-*Chd8*$^{+/-}$	0.71			
**P20 apical VGAT puncta (per 10 µm)**	M-WT vs M-*Chd8*$^{+/-}$	0.22	Interaction	(1, 44) = 4.69	0.04
	M-WT vs F-WT	0.27			
	F-WT vs F-*Chd8*$^{+/-}$	0.71			
	M-*Chd8*$^{+/-}$ vs F-*Chd8*$^{+/-}$	0.62			
**P20 basal VGAT puncta (per 10 µm)**	M-WT vs M-*Chd8*$^{+/-}$	0.002	Interaction	(1, 42) = 7.90	0.003
	M-WT vs F-WT	0.51			

	F-WT vs F-*Chd8*$^{+/-}$	> 0.99			
	M-*Chd8*$^{+/-}$ vs F-*Chd8*$^{+/-}$	0.06			
**P20 somatic VGAT puncta (per 10 µm)**	M-WT vs M-*Chd8*$^{+/-}$	0.80	Interaction	(1, 58) = 0.48	0.49
	M-WT vs F-WT	0.86	Sex	(1, 58) = 3.42	0.07
	F-WT vs F-*Chd8*$^{+/-}$	> 0.99	Genotype	(1, 58) = 0.27	0.61
	M-*Chd8*$^{+/-}$ vs F-*Chd8*$^{+/-}$	0.25			
